# Friends or foes: can we make a distinction between beneficial and harmful strains of the *Stenotrophomonas maltophilia* complex?

**DOI:** 10.3389/fmicb.2015.00241

**Published:** 2015-03-31

**Authors:** Gabriele Berg, Jose L. Martinez

**Affiliations:** ^1^Institute of Environmental Biotechnology, Graz University of Technology, GrazAustria; ^2^Centro Nacional de Biotecnologia – Consejo Superior de Investigaciones Científicas, MadridSpain

**Keywords:** *Stenotrophomonas maltophilia*, *S. rhizophila*, biocontrol, bioremediation, risk assessment

## Abstract

*Stenotrophomonas maltophilia* is an emerging multi-drug-resistant global opportunistic pathogen of environmental, mainly plant-associated origin. It is also used as a biocontrol or stress protecting agent for crops in sustainable agricultural as well as in bioremediation strategies. In order to establish effective protocols to distinguish harmless from harmful strains, our discussion must take into consideration the current data available surrounding the ecology, evolution and pathogenicity of the species complex. The mutation rate was identified as one of several possible criteria for strain plasticity, but it is currently impossible to distinguish beneficial from harmful *S. maltophilia* strains. This may compromise the possibility of the release and application for environmental biotechnology of this bacterial species. The close relative *S. rhizophila*, which can be clearly differentiated from *S. maltophilia*, provides a harmless alternative for biotechnological applications without human health risks. This is mainly because it is unable to growth at the human body temperature, 37^∘^C due to the absence of heat shock genes and a potentially temperature-regulated suicide mechanism.

## Introduction

In recent years, the number of human infections caused by opportunistic pathogens has increased dramatically. Plant organs, especially the rhizosphere (root) as well as the endosphere (inner tissues) are natural reservoirs of emerging opportunistic pathogens. Various bacterial genera including* Burkholderia, Enterobacter, Herbaspirillum, Ochrobactrum, Pseudomonas, Ralstonia, Staphylococcus*, and *Stenotrophomonas* contain plant-associated strains that can encounter dual interactions with both plant and human hosts (Parke and Gurian-Sherman, 2001; Berg et al., 2005). Opportunistic human pathogens with saprophytic phases or pathogens residing in environmental reservoirs, such as those provided by plants, are also referred to as environmental pathogens (Morris et al., 2009).

The mechanisms responsible for the colonization of plant and human tissues are similar (Berg et al., 2005). Further, multiple antibiotic resistances are not only found amongst clinical strains, but also within strains isolated from plants and often caused by multidrug eﬄux pumps (Berg et al., 1999; Martinez et al., 2009). High levels of competition, the occurrence of diverse antibiotics and secondary antimicrobial plant metabolites, and enhanced horizontal gene transfer and mutation rates in the microenvironment contribute to the development of high levels of natural resistance (Martínez, 2013; García-León et al., 2014). Congruently, these factors contribute favorably to the enormous potential application of these inhabitants as biocontrol or stress protecting agents in sustainable agricultural practices (Hirsch and Mauchline, 2012; Berg et al., 2013). Although recent research has elucidated the impact of pathogen ecology in environmental reservoirs on the evolution of novel or enhanced pathogen virulence (Morris et al., 2009), less is known about features that differentiate between pathogens and beneficial bacteria. Moreover, the question of how we can distinguish beneficial from harmful strains still remains, and it will be addressed here as it relates to the historical body of *Stenotrophomonas* research.

## Plant-Associated* Stenotrophomonas* Strains Occupy New Niches and Hosts

For a long time after its description in 1961 as *Pseudomonas maltophilia* (Hugh and Ryschenko, 1961), *Stenotrophomonas* belonged to the *Xanthomonadaceae*, a yellow pigmented bacterial family strongly associated with plants (Swings et al., 1983). Indeed, most of the species were able to cause diseases in plants, and in order to substantiate this taxonomic concept, the non-pathogenic *Xanthomonas maltophilia* was excluded from the genus. A new genus was established by Palleroni and Bradbury (1993). At that time, *Stenotrophomonas* was mainly known for its occurrence in plants, and many different plant species were reported as hosts including diverse crops, e.g., oilseed rape, maize, potato, cabbage, mustard, and beet (Juhnke and des Jardin, 1989; Berg et al., 1996). It was interesting to observe that especially in plants with extraordinary secondary metabolisms (*Brassicaceae*, eucalyptus) living in extreme habitats (dune environments) *Stenotrophomonas* belonged to the dominant bacterial inhabitants (De Boer et al., 2001; Ribbeck-Busch et al., 2005). Many of them showed an endophytic life style, representing a highly intimate interaction with its host (Krechel et al., 2002; Ryan et al., 2009). Many reports showed the enormous potential for agricultural biotechnology: strains of *Stenotrophomonas maltophilia* were able to promote plant germination and growth and to suppress plant pathogens (Berg and Ballin, 1994; Kobayashi et al., 1995; Nakayama et al., 1999; Dunne et al., 2000; Suckstorff and Berg, 2003; Messiha et al., 2007; Jin et al., 2011). *S. maltophilia* was used as an efficient biocontrol agent, and up until the 1980s, no significant risk to human health was reported. Use of the species in the decontamination of soil (bioremediation) has attracted considerable interest because of its capacity to degrade a wide range of xenobiotic compounds by a broad spectrum of unique enzymes (Binks et al., 1995; Ribitsch et al., 2012). Interestingly, *S. maltophilia* strains, e.g., OS4, was able to reduced silver nitrate (AgNO_3_) to generate cuboid and homogenous nanoparticles (AgNPs) with antimicrobial but without cytotoxic effects (Oves et al., 2013).

On the other hand*, S. maltophilia* has been reported since the early 1980s as a new pathogen in hospitals, and now it is a global pathogen and one of the most common opportunistic pathogens in hospitals (Ryan et al., 2009; Brooke, 2012). Although *S. maltophilia* does not usually infect healthy hosts (community infection), this bacterial species produce at hospitals bacteraemia and these infections are often associated with high mortality rates (reviewed in Brooke, 2012). *S. maltophilia* strains are characterized by multi-resistance to many antibiotics. In agreement with this situation, antibiotic treatment and the basal situation of the host (immunocompromised, cystic fibrosis) constitute the main risk factors for fatal *Stenotrophomonas* infections (Sanchez et al., 2009; Hernández et al., 2011; Brooke, 2012).

Due to their beneficial interactions with plants on one hand and on the other hand their facultative pathogenic interactions with humans, *Stenotrophomonas* strains challenge us to find differentiating features. Differentiation of features is of critical importance in terms of further applications in the field of biotechnology and in our understanding of the risks of infections and related epidemiological questions. The prediction of human health risks is currently one of the main challenges facing environmental biotechnology. The assessment of potential risk factors is the main obstacle in registration procedures, especially within the European Union (Ehlers, 2011).

## *Stenotrophomonas*: Diversity and Properties

*Stenotrophomonas maltophilia* was well-known for its intraspecific heterogeneity, which is already described in the type description by Palleroni and Bradbury (1993). This heterogeneity was confirmed in physiological studies at a phenotypic level (Swings et al., 1983; Van den Mooter and Swings, 1990) as well as at a genotypic level (Gerner-Smidt et al., 1995; Moore et al., 1997; Berg et al., 1999; Hauben et al., 1999). In the 1990s molecular fingerprinting methods were applied to distinguish species and find infection routes. These studies did not always result in clear conclusions, and in some of them a reservoir of infection was found in hospitals, e.g., in ice machines and in ventilators, however, other studies identified highly diverse strain patterns. By contrast, patient to patient transmission was found to be a rare event. In addition, antibiotic resistance patterns were monitored, and although the pattern varied, most of the analyzed strains were multi-resistant. It was originally assumed that multi-resistance was acquired in hospitals as *S. maltophilia* is naturally competent to acquire foreign DNA; however, strains isolated from plants also showed multiple antibiotic resistances (Berg et al., 1999). Multilocus sequence typing (Maiden, 2006) was applied for a highly diverse inter-continental selection of 70 *Stenotrophomonas* strains of various ecological origins (Kaiser et al., 2009). Interestingly, also in this study the heterogeneity was confirmed while on the other side geno-groups, which contained only isolates of strictly environmental including the *S. maltophilia* type strain, were identified. Gherardi et al. (2015) provide an overview of various typing methods including proteome-based bacterial identification using matrix-assisted laser desorption ionization-time of flight mass spectrometry (MALDI-TOF MS) for clinical epidemiology of *S. maltophilia* and suggest novel Web-based platforms for rapid data processing for outbreak investigations and surveillance studies in routine clinical practices.

We applied diverse typing strategies with the aim of differentiating clinically relevant strains from environmental ones (Berg et al., 1999; Minkwitz and Berg, 2001). From the 16S rDNA sequencing analysis, the isolates could be separated into three genomovars, two of which consisted of isolates originating from the environment (especially rhizosphere isolates; E1 and E2), and one which contained clinical and aquatic strains (C). In contrast to previous investigations (Denton and Kerr, 1998), most of the strains could be grouped according to their sources of isolation (Minkwitz and Berg, 2001), in spite of the fact that the antibiotic resistance profile of *S. maltophilia* isolates, and their ability to colonize plant roots, did not correlate with their origin (Suckstorff and Berg, 2003). However, it was possible to establish a new, clearly plant-associated species *S. rhizophilia* DSM14405^T^ (Wolf et al., 2002) with antagonistic activity toward fungal plant pathogens that comprised the isolates of the E1 cluster, and could be further distinguished from *S. maltophilia* isolates by (i) growth temperatures, (ii) xylose assimilation, and (iii) osmolyte production. Additionally, the *smeD* gene which is part of the genes coding for the multidrug eﬄux pump smeDEF from *S. maltophilia* was identified as a further genetic marker, (Ribbeck-Busch et al., 2005). In contrast to *S. maltophilia*, no pathogenicity to humans is known for plant-associated *S. rhizophila,* and fortunately there have been no reports of the species being associated with human infections or clinic environments to date.

## Can *S. rhizophila* Provide an Alternative for Biotechnological Applications?

*Stenotrophomonas rhizophila* is a species closely related both phylogenetically and ecologically to *S. maltophilia* (Wolf et al., 2002), and therefore a careful risk assessment for any biotechnological use is necessary. *S. rhizophila* is a model bacterium for a plant-competent, salt-tolerant plant growth promoting rhizobacterium (PGPR) with an endophytic lifestyle (Ryan et al., 2009; Berg et al., 2010). Plant growth promotion by the *S. rhizophila* strain DSM14405^T^ (synthesis strain e-p10) was observed under greenhouse conditions (Schmidt et al., 2012) and in the highly salinated soils of Uzbekistan (Egamberdieva et al., 2011). The differences between *S. maltophilia* and *S. rhizophila* were analyzed by comparative genomics (Alavi et al., 2014). Despite the notable similarity in potential factors responsible for host invasion and antibiotic resistance, other factors including several crucial virulence factors and heat shock proteins were absent in the plant-associated *S. rhizophilia* DSM14405^T^. Instead, *S. rhizophila* DSM14405^T^ possessed unique genes responsible for the synthesis and transport of the plant-protective spermidine, plant cell-wall degrading enzymes, and high salinity tolerance. In addition, spermidine and osmoprotectant production (glucosylglycerol and trehalose) was the main response of *S. rhizophilia* DSM14405^T^ to rhizosphere exudates in a transcriptomic study, which suggested the involvement of these substances in the mode of interaction with plants (Alavi et al., 2013b). Moreover, the capability of bacteria for growing at 37^∘^C was identified as a very simple criterion in differentiating between pathogenic and non-pathogenic *S. maltophilia* and *S. rhizophila* isolates. DSM14405^T^ is not able to grow at that temperature, most likely in great part due to the absence of heat shock genes and perhaps also because of the up-regulation at increased temperatures of several genes involved in a suicide mechanism (Alavi et al., 2014). The conclusion of these studies is that *S. rhizophila* currently does indeed provide an alternative to biotechnological applications without posing any risks to human health. The main reason for this conclusion is the demonstration of a lack of any growth at 37^∘^C and the identified underlying mechanisms, which should prevent or disallow colonization of the human body. It was suggested that *S. rhizophila* can be used as a biocontrol and stress protecting agent (Alavi et al., 2013b), however, as we have learned, host–microbe interaction is a co-evolutionary process, and the outcome of interaction can be changed by many factors, which suggest a need for preventive genomic monitoring.

## An Evolutionary Concept to Explain *Stenotrophomonas* Diversity and Plasticity

Within three decades *S. maltophilia* developed from a typical plant-associated species into a serious human pathogen. How was this possible? *S. maltophilia* belongs to the bacterial group of *r*-selected species that places an emphasis on a high growth rate, and typically exploiting less-crowded ecological niches producing a high number of bacteria in a short time.

In contrast to the reported heterogeneity of the *S. maltophilia* complex, the diversity of pathogenicity and interaction factors seems to be low. Proteases, siderophores, and biofilm formations are reportedly regulated by the DSF quorum sensing system (Alavi et al., 2013a). Multiple eﬄux systems are responsible for the resistance to antibiotics, toxins, and metals (Ryan et al., 2009). Anbitiotics and volatiles were shown to be involved in the anti-eukaryotic activity (Jacobi et al., 1996; Kai et al., 2007). However, clinical and environmental *S. maltophilia* strains presented comparable distribution of identified potential virulence genes thus far (Adamek et al., 2014) and they harbor the same resistance determinants, which make them highly resistant to antibiotics currently in use at clinical settings.

Altogether, this suggests that the main reason for the recent emergence of *S. maltophilia* as a relevant pathogen may reside in the host itself more than in a process of bacterial evolution. As above stated, *S. maltophilia* is a prototype of highly resistant microorganism. Debilitated patients at hospitals are more prone to infection than healthy people. In this situation, the main factor impeding infection is antibiotic prophylaxis or treatment. In this situation of high antibiotic load, organisms highly resistant as *S. maltophilia* should have higher chances for surviving and hence produce infection. Virulence factors and resistance elements, likely acquired in the field for plant colonization may have allowed *S. maltophilia* to become an infective bacterium, just in debilitated patients with underlying diseases.

This does not mean, however, that *S. maltophilia* cannot further evolve during infection. These bacteria are characterized by a high rate of genomic re-arrangements and hypermutator activity, which allow rapid adaptation to new niches. We were able to confirm the latter for *S. maltophilia* strains: clinical strains belonged exclusively to the hypermutators, whereas environmental strains showed a broader spectrum of mutation rates (Turrientes et al., 2010). This indicates that the mutation rate is an important criterion of assessing the probability that a bacterial strain can occupy new niches and hosts. The rate and effects of mutations is one of the main ecological and genetic factors that may affect the likelihood of emergence of a pathogen (Gandon et al., 2013). In a long-term study analyzing *S. maltophilia* strains from chronically colonized cystic fibrosis patients, Vidigal et al. (2014a) was able to demonstrate that different genotypes with different mutation rates including 31.2% strong hypermutators exist. As a sign of adaption their mutation status switches over time to a less mutator phenotype without increasing resistance, which suggests that *S. maltophilia* attempts to sustain its biological fitness as a mechanism for long-term persistence. Horizontal gene transfer is another mechanism by which pathogenicity islands can be acquired. The natural capacity for DNA uptake of *S. maltophilia* was shown by strains carrying pathogenicity island from other species, e.g., from *Staphylococcus aureus* (Ryan et al., 2009). All of these factors contribute to the high intra-specific heterogeneity and genomic plasticity of *Stenotrophomonas,* which allow them not only to colonize new hosts but also to develop new genotypes and species.

## Conclusion

The current established theory of opportunistic pathogens is that the ancestors of virulent bacteria as well as the origin of virulence and resistance determinants are most likely to originate from environmental microbiota (Martínez, 2013). *S. maltophilia* is an appropriate model, which fits into this theory. It is currently impossible to distinguish between beneficial and harmful *Stenotrophomonas* strains. This evidence may compromise the possibility of any application for environmental biotechnological purposes. Although bacterial strains adapt to its specific niches and then develop new properties, *S. rhizophila* has been clearly differentiated from the *S. maltophilia* complex and seems not to pose a risk.

## Future Aspects

In recent years, we have deciphered parts of a puzzle in our efforts to understand the evolutionary process within the *Stenotrophomonas* complex. We have gained new insights into the lifestyle, the physiology, and potential risk factors as antibiotic resistance elements or virulence determinants of *Stenotrophomonas* through the application of genomics and transcriptomic techniques. Having identified a completely different methylation rate for different *Stenotrophomonas* strains, interpretation of epigenetic analyses is the current challenge that we are confronted with. This opens up new possibilities for the manipulation of the bacteria, while also requiring an understanding of the physiology and ecology of the bacteria. However, there are still taxonomical problems to be solved, problems which led to misidentifications in the past as in the case of the perceived overlap between *Stenotrophomonas* and *Lysobacter*. The latter is also a biotechnologically relevant genus because of its micropredatory activity (Hayward et al., 2009). Brooke (2014) reviewed new strategies for prevention and treatment of *Stenotrophomonas* infections in great detail. Therefore, we would like to suggest alternative strategies which require a thorough rethinking of the entire approach to microbiome management (Arnold, 2014). *Stenotrophomonas* strains closely interact with phages; they carry different phages in their genome but lytic phages have also been identified (Hagemann et al., 2006; García et al., 2008). This may explain a fast evolution by selection pressures on bacteria and open new possibilities for phage therapies for multi-resistant *Stenotrophomonas* (Vos et al., 2009). Maltocin P28, the first identified phage tail-like bacteriocin from *S. maltophilia* is a promising therapeutic substitute for antibiotics for *S. maltophilia* infections (Liu et al., 2013). In addition to phages, probiotic bacteria demonstrating antagonistic activity toward *S. maltophilia* would be an interesting alternative to consider in the prevention of infections. Natural products like the green tea compound epigallocatechin-3-gallate have also showed a promising activity against *S. maltophilia* (Vidigal et al., 2014b).

## Conflict of Interest Statement

The authors declare that the research was conducted in the absence of any commercial or financial relationships that could be construed as a potential conflict of interest.
